# Case Report: Ablation as novel x therapy in the regimen for frail hematological malignancies with extramedullary lesion

**DOI:** 10.3389/fonc.2025.1723537

**Published:** 2025-11-10

**Authors:** Muyun Wu, Shanshan Shi, Yan Guo, Manya Yu, Runjie Sun, Mengting Xia, Ye Yang, Xing Cui

**Affiliations:** 1 First Clinical Medical College of Shandong University of Traditional Chinese Medicine, Jinan, China; 2 Department of Healthcare, Qilu Hospital of Shandong University, Jinan, China; 3 Department of Hematology, The First Affiliated Hospital of Shandong First Medical University and Shandong Provincial Qianfoshan Hospital, Jinan, China; 4 Department of Oncology and Hematology, Second Affiliated Hospital of Shandong University of Traditional Chinese Medicine, Jinan, China; 5 School of Medicine, Nanjing University of Chinese Medicine, Nanjing, Jiangsu, China

**Keywords:** myeloma, lymphoma, bulk, relapsed, refractory, frail, ablation, extramedullary

## Abstract

Hematological diseases, as a category of malignant tumors originating from the hematopoietic system, have long relied on treatments such as chemotherapy, radiotherapy, and hematopoietic stem cell transplantation. However, challenges remain in managing locally progressive lesions, relapsed/refractory lesions, and involvement of special anatomical sites, especially in infirmities. Ablation technology, as a minimally invasive local treatment method, directly destroys lesion tissues through physical energy and has been widely used in the field of solid tumors. In recent years, with advancements in image-guided technology and the presence of unmet clinical needs in the local treatment of hematological diseases, studies have begun to explore the application of ablation technology in hematological diseases. We retrospectively analyzed 4 frail hematological malignancies cases to discuss the application significance and indications of local ablation therapy in hematological diseases of frail patients, aiming to provide references for clinical practice.Case 1 and Case 2 involved patients with relapsed/refractory multiple myeloma. Both patients had extramedullary lesions in the left maxillary region and the left tibial anterior area, and were unable to tolerate intensive treatment. Case 3 was diagnosed with diffuse large B-cell lymphoma (DLBCL) combined with large extramedullary lesion masses. Case 4 was diagnosed with DLBCL combined with synovial sarcoma. Case 3 and 4 patients were over 80 years old. The four patients were treated with a combination of local ablation and low-dose systemic chemotherapy. After the treatment, all four patients achieved varying degrees of remission, improvement in local compression symptoms, and enhancement of quality of life, with no obvious side effects. Our study demonstrated for the first time the good safety and efficacy of ablation combined with chemotherapy for frail patients, and also indicated that ablation can be used as a new treatment strategy for elderly frail B-cell malignancies patients with local extramedullary lesions who cannot tolerate intense systemic treatment, or for those B-cell malignancies with multiple relapses.

## Introduction

Treatments for lymphomas and multiple myeloma (MM) in malignant hematological diseases mainly include chemotherapy, immunotherapeutic agents, and hematopoietic stem cell transplantation. Although these treatments have significantly improved patient prognosis, challenges such as limited efficacy and significant toxic side effects remain for locally progressive lesions—especially in frail patients with bulky masses, relapsed/refractory lesions, or involvement of special anatomical sites. Compared to MM patients without extramedullary lesions, the management of extramedullary MM (EMM) is challenging (1, 2). The presence of EMM with a single lesion exceeding 5 cm in diameter or with more than two lesions is a poor prognostic factor for MM. Although some agents and combinations have shown a degree of efficacy, their effectiveness is generally lower than in patients without extramedullary involvement (3).Existing evidence indicates that bulky masses are one of the key factors contributing to poor prognosis in diffuse large B-cell lymphoma (DLBCL) (4, 5).For the local treatment, a significant advantage to adding involved-site radiation therapy (ISRT) following complete remission (CR, evaluated by CT criteria) for extranodal involvement was observed (6). Current systemic treatment regimens mainly focus on “ + novel mechanism agents(X)” such as Pola-R-CHP, CAR-T therapy, and selinexor.

Old and frail patients, due to their inability to tolerate the standard dose of treatment, did not achieve satisfactory results. According to the International Myeloma Working Group (IMWG) frailty score, the 3-year overall survival (OS) rates of myeloma patients were 84% in the healthy group, 76% in the moderately frail group, and 57% in the frail group, the main challenge in treating frail MM patients—especially those with EMM—is their limited tolerance to intensified treatment (7). The European Hematology Association (EHA) Guidelines state that patients aged 80 years or older, or those under 80 years with poor physical status (8). For patients with good physical status, R-mini-CHOP may be substituted for very frail patients and patients >80 years of age with comorbidities to improve tolerability (9–11).

As a result, there is a need to incorporate localized therapies into the treatment regimen. Ablation is a minimally invasive procedure that includes thermal ablation, such as radiofrequency ablation (RFA) and microwave ablation (MWA), as well as cryoablation. It has been extensively used to treat a variety of solid tumors, including liver cancer, lung cancer, breast cancer, and prostate cancer (12). In MM, it is mainly used for the treatment of myeloma bone disease (13). Ablation has also been sporadically reported in the treatment of lymphoma (14).Ablation can quickly control local tumor progression, reduce pain, and maintain local tissue integrity, especially for treating tumors ≤ 3 cm (15, 16). However, the use of ablation combined with systemic therapy in frail hematological malignancies has not yet been reported. Therefore, this study aimed to explore the safety and efficacy of this combination treatment.

## Patient case 1

**Figure 1:**
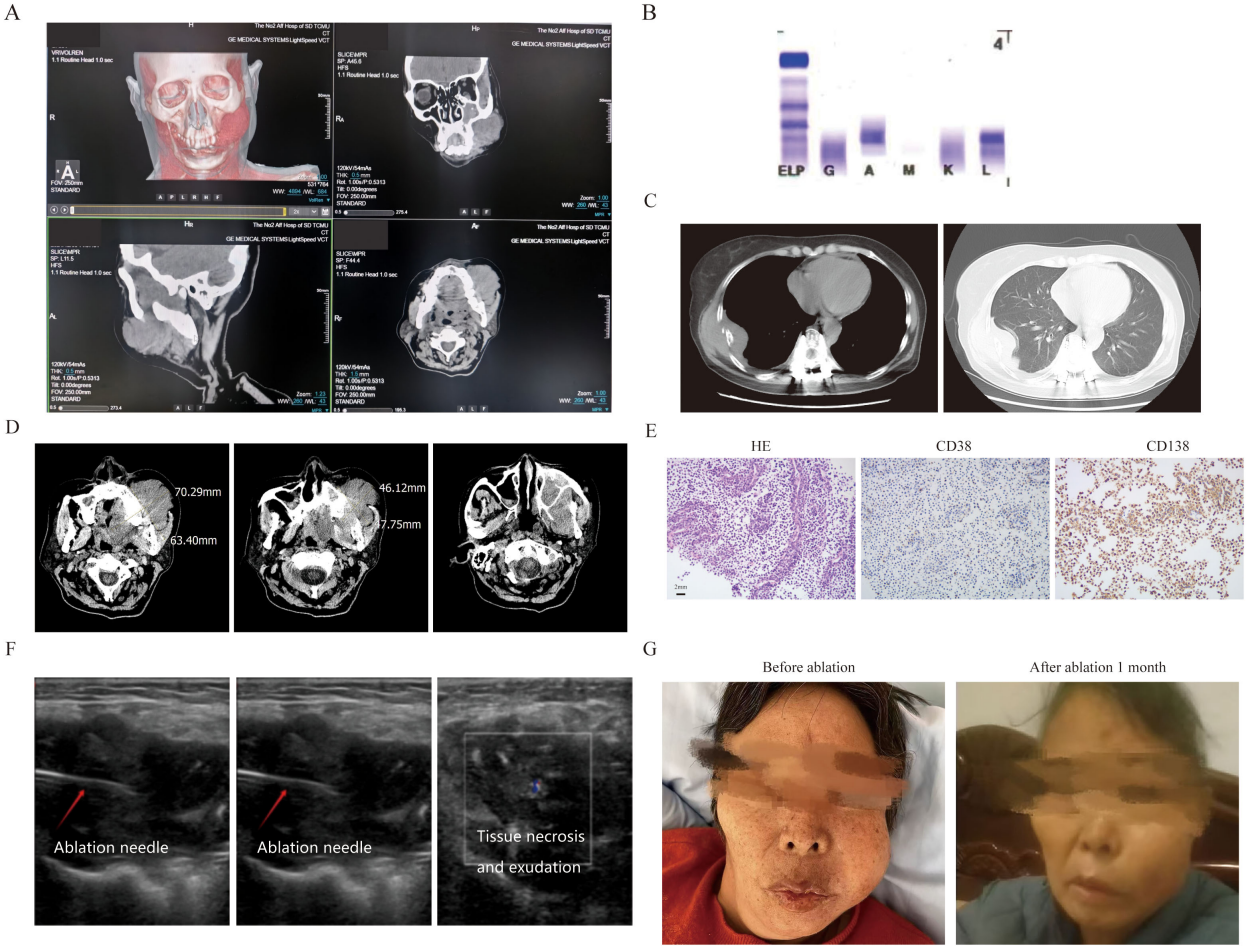
The clinical data of patient 1. **(A)** Three-dimensional CT imaging of the left face. **(B)** The immunofixation electrophoresis image. **(C)** The extramedullary lesion beside the rib. **(D)** The extramedullary lesion on the left side of the face scanned by CT before ablation treatment. **(E)** The pathological image of the extramedullary lesion on the left face(20×original magnification). **(F)** The process of microwave ablation under color ultrasound guidance. The first picture shows the position where the ablation needle reached, the second picture shows the start of the ablation process, and the third picture shows the moment after the ablation is completed and the ablation needle has been withdrawn. **(G)** The extramedullary lesion on the left side face before and after microwave ablation treatment.

The first patient was a 56-year-old female who was diagnosed as MM(IgA-λ, DS III stage, ISS II stage, R-ISS II stage). Then she was treated with 8 cycles of VRD regimen and was evaluated as having achieved a very good partial response (VGPR). Due to personal reasons, she did not undergo autologous hematopoietic stem cell transplantation and was given lenalidomide 25 mg orally once daily for maintenance treatment. She had a relapse one year later, and was treated with the KPD regimen. After 4 cycles, she developed severe swelling and pain in the left maxillofacial area, was unable to close her mouth, and experienced a fever (**Figure 1A**). OxyContin 30 mg administered every 12 hours failed to provide adequate analgesia. Clinical test showed disease progression, and she couldn’t tolerate intensive chemotherapy: hemoglobin:105g/l, platelet:70×10^9^/l, M protein level: 1.2g/l, M protein percentage: 1.9% (**Figure 1B**). A sinus tract approximately 1.7 cm in width was observed extending into the deep tissues.Biopsy results of her mouth revealed abnormal plasma cells in the puncture tissue. CT scan demonstrated bone destruction (**Figure 1C**), the EMM lysion of mouth also can be confirmed on CT (**Figure 1D**) .Immunohistochemistry (IHC) showed that the tumor cells were positive for CD38, CD138, and MUM1, with a Ki-67 proliferation index of approximately 40% (**Figure 1E**). A frailty assessment performed that an ECOG score of 3 and an ADL score of 65; the results indicated an activities of daily living score of 1, independent activities of daily living score of 2, and a Charlson Comorbidity Index level of 6. After multidisciplinary assessment, the patient was deemed eligible for MWA. The MWA instrument (MWD-A; Reebo Medical, Nanjing, China) was used to administer microwave energy. According to ultrasound guidance, a 16G ablation needle was inserted into the interior of the mass. Two cycles of ablation were performed, each at a power of 50 W for 10 minutes. When the ablation completely covered the hyperechoic area, Contrast - Enhanced Ultrasound(CEUS)confirmed that there was no enhancement of the ablation area, indicating that the ablation was completed (**Figure 1F**). The patient was instructed to apply ice after ablation. No significant adverse events were observed except for minor bleeding. Postoperatively, the patient could close her mouth, and the pain was tolerable without the need for regular analgesics. After one week, the patient recovered well and was discharged with an ECOG score of 2 and an ADL score of 85. **Figure 1G** showed her mouth after ablation treatment 1 month.But unfortunately, she gave up further treatment due to financial reasons and passed away three months later.

## Patient case 2

**Figure 2:**
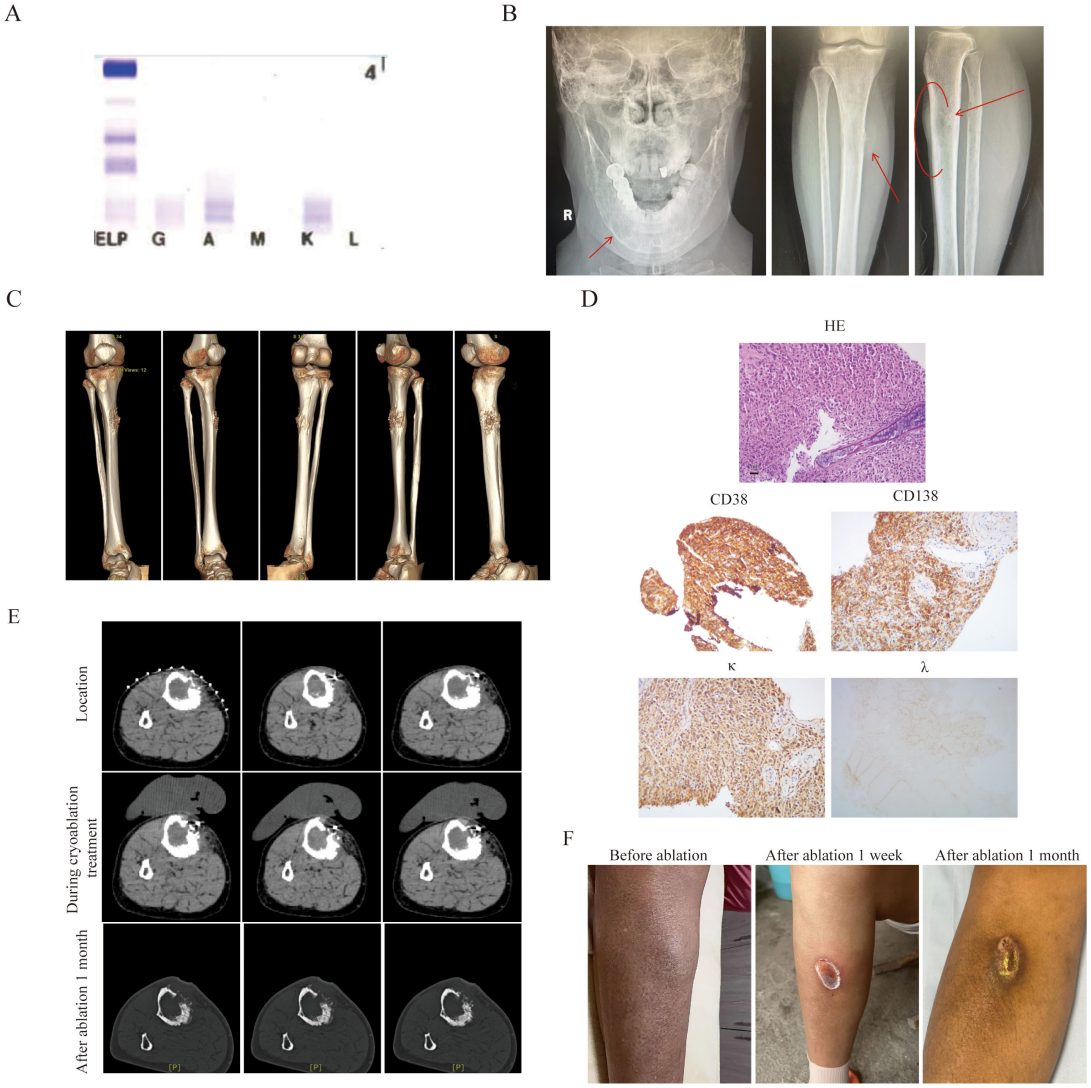
The clinical data of patient 2. **(A)** The immunofixation electrophoresis image. **(B)** The extramedullary lesion of mandible and anterior side of the tibia. **(C)** Three-dimensional stereoscopic imaging of extra-membranous lesions of the tibia. It can be observed that this extramedullary lesion grows out from the bone cortex. **(D)** The pathological image of the extramedullary lesion(20×original magnification). **(E)** The extramedullary lesion CT scan pictures before and after cryoablation treatment.To prevent frostbite, insulating pads were used on the skin during cryoablation. **(F)** The tibia pictures before and after ablation treatment.

The second patient was a 55-year-old female who diagnosed as MM(IgA κ, ISS II stage R-ISS II stage, R-ISS κ stage) (**Figure 2A**) two year ago. Then she was treated with 8 cycles of VRD regimen and was evaluated as having achieved CR. Due to personal reasons, she did not undergo autologous hematopoietic stem cell transplantation and was given lenalidomide 25 mg orally once daily for maintenance treatment. She experienced a relapse one and a half years later, with a painful 3 × 5 cm mass adjacent to the right tibia (**Figures 2B, C**). Her IMWG frailty score was 2 with an ECOG score of 3 and an ADL score of 60, classifying her as frail. A needle biopsy of the anterior right tibial mass revealed myeloma cells. IHC showed CD38 (+), CD138 (+), MUM (+), CD79α (+), kappa (+), Ki-67 (+, 40%), CK (-), CD20 (-), and lambda (-) (**Figure 2D**). Then, she was treated with the KPD regimen. After two cycles, new bulges appeared on the left forehead and around the eye, suggestive of EMM, and the mass in the right leg had not shrunk (**Figure 2A**). Therefore, cyclophosphamide (600 mg on days 1, 8, and 15) was added to the regimen. Following two additional cycles, the extramedullary lesions showed slight reduction, but the patient developed severe bone marrow suppression, with neutrophils at 0.54 × 10^9^/L, hemoglobin at 75 g/L, platelets at 92 × 10^9^/L, and fever. After multidisciplinary assessment, the patient’s treatment regimen was adjusted to reduced cyclophosphamide (600mg on d1 and 8), combined with CT-guided percutaneous cryoablation for the mass in the right leg to reduce tumor burden (**Figure 2D**). Cryoablation was performed using an Argon-Helium Cryosurgical System (AH-22, Beijing Sunlight Yi Bang Medical Science and Technology Co., Ltd.). A 17G cryoprobe was inserted into the mass under CT guidance, and two freeze-thaw cycles were conducted, each consisting of 8 minutes of freezing followed by 2 minutes of thawing (**Figure 2E**). Warm pads were applied to minimize skin injury caused by freezing. No serious adverse events occurred during the ablation procedure. Following ablation, pain in the right leg was relieved, and myeloma response assessment indicated a VGPR with an ECOG score of 2 and an ADL score of 90.One month later, the lesion was basically healed (**Figure 2F**).

## Patient case 3

**Figure 3:**
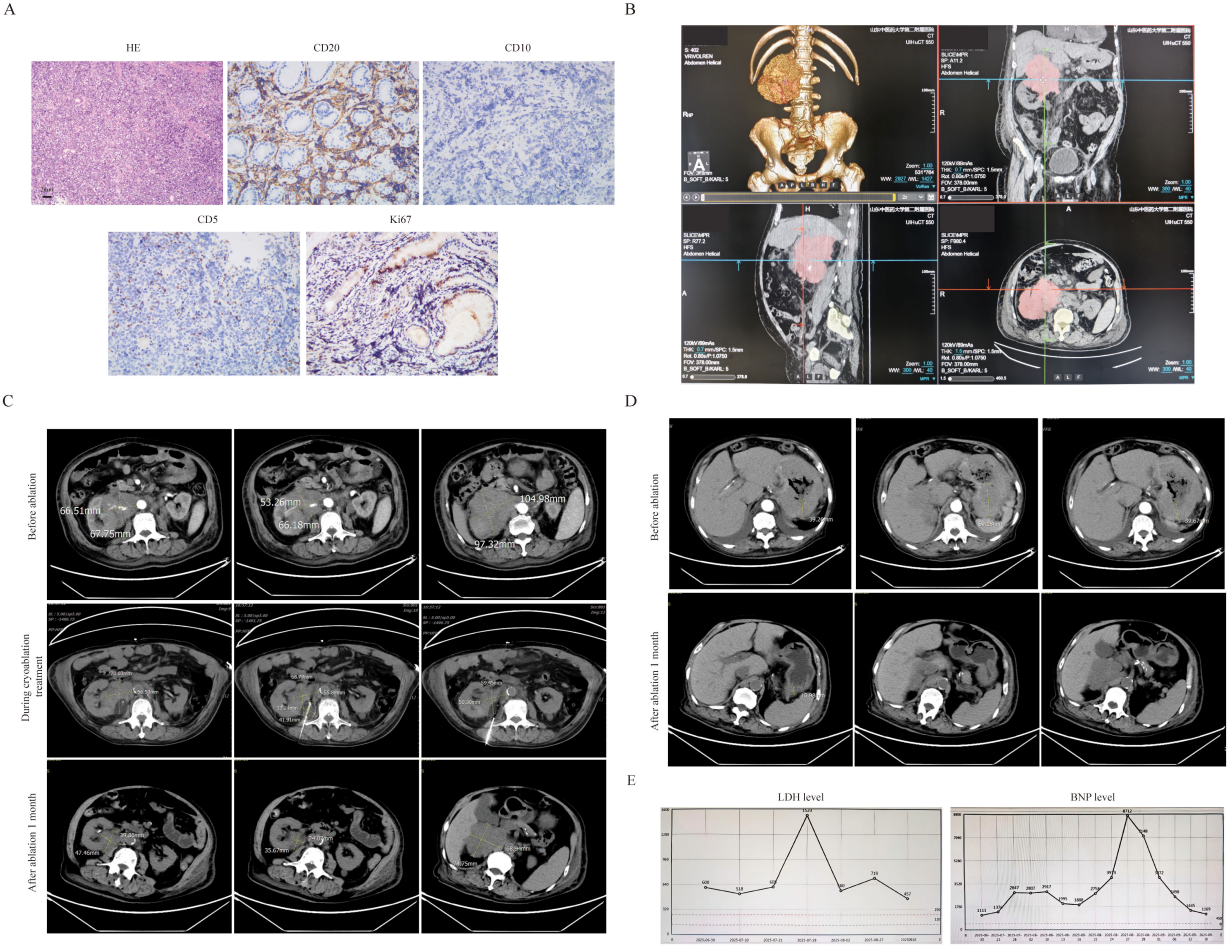
The clinical data of patient 3. **(A)** The pathological image of retroperitoneal mass(20×original magnification). **(B)** CT 3D reconstruction. **(C)** The retroperitoneal bulk before and after cryoablation treatment .It can be clearly seen that the volume of the ablated mass has significantly decreased(2.4×3.5cm vs 5.5×6.8cm). **(D)** After ablation treatment, the thickness of the stomach wall has also significantly decreased(1.5cm vs 6.9cm). **(E)** After ablation treatment, the level of LDH(457 vs 1523) and BNP(1169 vs 8712) have also significantly decreased.

The third patient was an 83-year-old male diagnosed by gastroscopic pathology as diffuse large B-cell lymphoma (DLBCL, Stage IV, high-risk with an International Prognostic Index(IPI) score of 5). The key challenge in this patient’s treatment was a large retroperitoneal mass measuring 9.1 cm × 9 cm, which encased the renal artery and caused renal compression (**Figure 3A**). Laboratory tests revealed a lactate dehydrogenase (LDH) level of 1523 U/L and a B-type natriuretic peptide (BNP) level of 8712 pg/mL. The patient required prolonged bed rest, was unable to walk independently, had an Eastern Cooperative Oncology Group (ECOG) performance status score of 4, and an Activities of Daily Living (ADL) score of 20.After the pathology of retroperitoneal mass was confirmed as also DLBCL (**Figure 3B**), treatment was administered as the R-miniCHOP regimen combined with cryoablation of the retroperitoneal mass. Under CT guidance, a 24G cryoprobe was inserted into the mass, and two freeze-thaw cycles were conducted using Argon-Helium Cryosurgical System—each cycle consisting of 15 minutes of freezing followed by 3 minutes of thawing. The ablation zone included a 5–10 mm margin around the targeted mass (**Figure 3C**). No serious adverse events occurred during the ablation procedure. After 2 cycles of R-miniCHOP, the treatment response was evaluated as partial remission (PR). The size of the retroperitoneal mass was 4.7×3.9cm (**Figure 3C**), the thickness of the stomach wall also decreased from 6.9 cm to 1.5 cm (**Figure 3D**), the LDH level decreased to 457 U/L, and the BNP level dropped to 1169 pg/mL (**Figure 3E**). The patient’s frailty improved significantly, with an ECOG score of 3 and an ADL score of 60.

## Patient case 4

**Figure 4:**
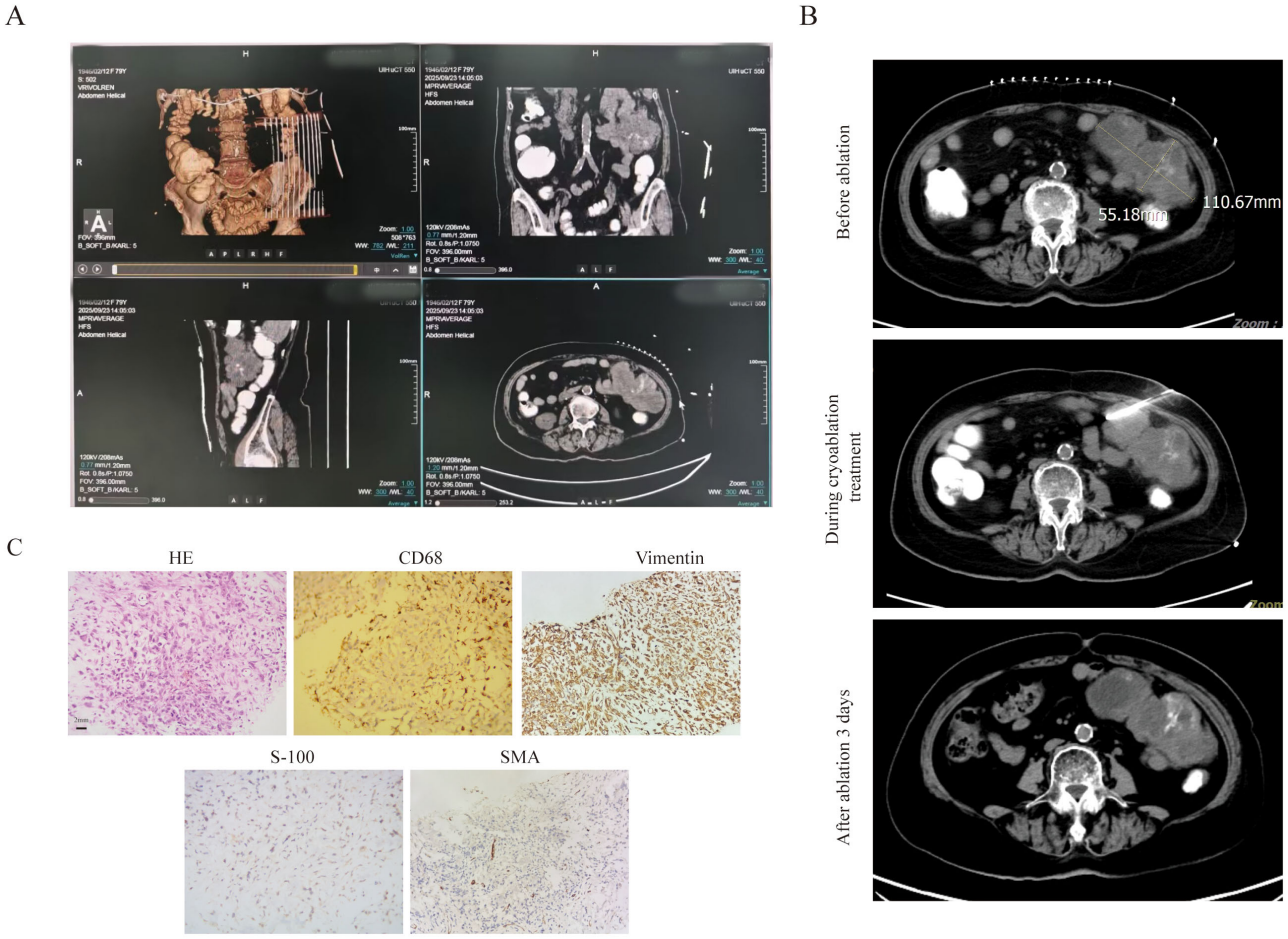
The clinical data of patient 4. **(A)** To clarify the relationship between the abdominal mass and the ascending colon, three-dimensional stereoscopic imaging of abdominal masses was performed. **(B)** The bulky disease CT scan pictures before and after cryoablation treatment. **(C)** The pathological image of the bulk(20×original magnification).

The fourth patient was an 80-year-old female diagnosed with DLBCL (Stage IVB). On November 22, 2024, lymph node aspiration biopsy confirmed DLBCL of the germinal center subtype. Immunohistochemical (IHC) results showed: CD20 (+), CD5 (+), CD10 (-), MUM-1 (focal +, <10%), Bcl-6 (+, >30%), Bcl-2 (+), CD3 (focal +), CD21 (-), P53 (30%), CD30 (rare +, <1%), C-MYC (30%), Ki-67 (80%); *in situ* hybridization (ISH) revealed EBER (-). Flow cytometry of bone marrow aspiration detected 1.09% monoclonal B lymphocytes. The patient’s treatment history was as follows: One cycle of R-CHOP (rituximab-CHOP), five cycles of zuberituzumab-CHOP administered, one cycle of Pola-BR. The patient achieved CR after the above treatments and was subsequently placed on maintenance therapy with oral lenalidomide. In early September 2025, the patient developed a left abdominal mass accompanied by distending pain. CT scans showed multiple lymph nodes in the abdominal cavity and retroperitoneum, with the largest lesion located on the left side of the abdomen (measuring 10.8 cm × 6.1 cm). The mass contained patchy low-density areas and punctate high-density areas, with a slightly larger range than in previous scans, and was closely associated with surrounding intestinal loops. Enhanced CT also showed positivity in some lymph nodes, suggesting disease recurrence. On September 9, the patient was initiated on Pola-BR + selinexor therapy. Considering the patient’s advanced age, frailty, significant myelosuppression (neutropenia and thrombocytopenia) from prior chemotherapy (rendering her unable to tolerate more intensive regimens), and the risk of radiation enteritis with radiotherapy to this region, cryoablation of the intra-abdominal mass was combined with systemic therapy. Before treatment, the patient had an ECOG score of 3 and an ADL score of 40.Details of the cryoablation procedure: First, contrast agent was administered orally 1.5 hours before ablation, then CT 3D reconstruction was used to further clarify the anatomical relationship between the intestinal tract and the mass (**Figure 4A**). Prior to ablation, tissue biopsy was performed, and approximately 30 ml of liquefied necrotic fluid was aspirated from the target area. Then, under CT guidance, a 24G cryoprobe was inserted into the mass, and two freeze-thaw cycles were conducted—each cycle consisting of 15 minutes of freezing followed by 3 minutes of thawing (**Figure 4B**). After treatment, the patient’s pain was significantly relieved, with an ECOG score of 2 and an ADL score of 65. Subsequent pathological examination of the intra-abdominal mass confirmed synovial sarcoma. IHC results for the synovial sarcoma were: Vimentin (+), EMA (+), CK (+), CD68 (+), CD10 (+), bcl-2 (focal cytoplasmic +), CD117 (-), Dog-1 (-), CD34 (vascular +), S100 (-), SMA (-), desmin (-), WT-1 (-), CK5/6 (-), D2-40 (-), and a Ki-67 proliferation index of 70% (**Figure 4C**). A combined regimen targeting both lymphoma and synovial sarcoma—Pola-R-CHP —was administered in combination with local cryoablation. After 1 cycle of this treatment, the patient’s condition improved further.

## Discussion

Systemic therapy serves as the primary treatment for EMM. Clinical studies have indicated that agents including carfilzomib (17), pomalidomide (18), daratumumab (19), and Chimeric Antigen Receptor T-Cell (CAR-T) therapy (20) all exert certain effects in the treatment of EMM. They can also be combined with novel mechanism drugs such as Selinexor to form new treatment regimens (21). A similar situation exists in the treatment of bulky lymphoma masses; for instance, Selinexor (22) and Polatuzumab (23) are used clinically. Although studies such as the HOVON HO143 study (24), ASPIRE study (25), ARROW trial (26), ALCYONE trial (27) and R-mini-CHOP treatment for lymphoma (28–30) have demonstrated the favorable tolerability, for those patients with lymphoma and large masses who are recognized as a distinct group with poor survival outcomes (35–37), there is still no systemic treatment regimen with relatively high efficacy for frail patients with extramedullary myeloma or lymphoma with bulky disease. Moreover, radiotherapy can be considered for solitary plasmacytoma (31–34) or bulky lymphoma (38–40).

Patients in Cases 1 and 2 were diagnosed with relapsed/refractory multiple myeloma (RRMM) complicated by EMM and were classified as frail. The most urgent issue of patient in Case 1was a mass in the left maxillofacial region, which caused severe pain and prevented the patient from closing her mouth, seriously compromising her quality of life. The patient in Case 2 had also undergone multiple lines of chemotherapy, and her extramedullary lesions responded to the KPCD regimen. However, the development of grade 3 bone marrow suppression. Given the two patients’ intolerance to intensified treatment, ablation was used in combination with low-dose systemic chemotherapy, this approach would minimally impact the patient’s lifespan and quality of life.

Patients in Cases 3 and 4 were diagnosed with DLBCL complicated by bulky disease and were both ≥80 years old. The patient in Case 3 faced issues of retroperitoneal mass compression on the kidneys, leading to edema and heart failure, accompanied by extremely high lactate dehydrogenase (LDH) levels. The patient’s condition was extremely poor and unable to tolerate intensive chemotherapy, while R-mini-CHOP was also ineffective in addressing the high tumor burden. Therefore, cryoablation was used in combination with R-mini-CHOP chemotherapy to reduce the local tumor burden, relieve the compression of the kidneys and renal arteries, resolve the edema, and effectively control the BNP levels associated with heart failure. The patient in Case 4 had received multiple lines of chemotherapy. Considering that the patient had experienced bone marrow suppression and severe infection during treatment with R-CHOP, Pola-BR combined with Selinexor, the patient could not tolerate further intensified treatment. Additionally, the patient’s intra-abdominal mass was adjacent to the descending colon, causing abdominal pain and poor appetite. Therefore, on the basis of Pola-R-CHP treatment, local cryoablation was added. After the completion of treatment, the patient’s gastrointestinal function improved to a certain extent.

Based on our series cases and other studies, we can indicate that, compared with systemic treatments (chemotherapy, targeted Therapy, etc.), the ablation has these advantages (1):Precise local efficacy. Directly acts on lesions via physical energy (heat, cold, etc.), causing less damage to normal tissues and reducing systemic toxic side effects (e.g., myelosuppression, gastrointestinal reactions). Particularly suitable for elderly, frail patients or those with multiple organ dysfunction (41) (2).Reduced tumor burden. Eliminates local residual lesions resistant to chemotherapy, creating conditions for subsequent systemic treatments (e.g., CAR-T therapy) (42, 43). (3)For masses that compress vital organs (44), severe bone lesions causing severe pain (especially in the spine, pelvis, etc.) (45, 46), and even situations where other key organs such as the eyes are affected and their functions impaired (42), these treatments can rapidly reduce the lesions, relieve pain and improve the quality of life.(4) Compared with Radiotherapy, ablation can provide superior minimal invasiveness without radiation exposure. Ablation can be performed multiple times, and under image guidance, the ablation range can be dynamically adjusted to accurately avoid adjacent sensitive tissues. The ablation has also some disadvantages:(1)Limited scope of action.(2)Ineffective for diffuse lesions (e.g., extensive bone marrow involvement). (3) For large-sized or irregularly bounded lesions, the local recurrence rate after ablation may be higher than that of radiotherapy (especially high-dose radiotherapy).

## Conclusion

Ablation combined with systemic chemotherapy can reduce tumor burden by locally inactivating lesions, enhance the sensitivity of chemotherapy to residual lesions, and facilitate deep disease remission—with significant effects especially on local bulky masses or residual lesions resistant to chemotherapy. For elderly and frail patients, this regimen can reduce chemotherapy doses, lower toxic side effects such as myelosuppression and gastrointestinal reactions, and improve treatment tolerance. In palliative care, ablation can quickly shrink lesions compressing critical organs to relieve symptoms like pain, while low-dose chemotherapy controls systemic progression, balancing efficacy and quality of life. Future efforts should include more clinical studies to optimize the timing of combination and ablation parameters and provide more precise individualized regimens for refractory cases and elderly patients.

## Data Availability

The raw data supporting the conclusions of this article will be made available by the authors, without undue reservation.
